# Psychological Well-Being of Children with Asthma and Their Parents

**DOI:** 10.3390/jcm13175100

**Published:** 2024-08-28

**Authors:** Valentina Agnese Ferraro, Silvia Spaggiari, Stefania Zanconato, Letizia Traversaro, Silvia Carraro, Daniela Di Riso

**Affiliations:** 1 Unit of Pediatric Allergy and Respiratory Medicine, Women’s and Children’s Health Department, University of Padova, 35128 Padova, Italy; stefania.zanconato@aopd.veneto.it (S.Z.); letizia.traversaro@gmail.com (L.T.); silvia.carraro@unipd.it (S.C.); 2 Department of Developmental and Socialization Psychology (DPSS), University of Padua, 35131 Padova, Italy; silvia.spaggiari@phd.unipd.it (S.S.); daniela.diriso@unipd.it (D.D.R.)

**Keywords:** asthma clinical aspects, asthma disease management, asthma risk factors, pediatric asthma, psychological well-being, psychology

## Abstract

**Background/Objectives**: The literature reports that emotional disorders in asthmatic children and their parents may affect asthma control. This research explores the baseline psychological well-being of asthmatic children and their mothers’ and fathers’ psychological functioning, focusing on the influence of the parents’ anxiety and separation anxiety on children’s asthma. **Methods**: In this cross-sectional study, we enrolled children with non-severe asthma and their parents, and a group of healthy children and their parents. The asthmatic children underwent anamnestic investigation, including asthma control and spirometry. Then, both the asthmatic and control triads filled a survey assessing their psychological functioning. **Results**: Among the 40 asthmatic children enrolled (8–18 years old), most had good clinical control maintained with GINA (Global Initiative for Asthma) therapy step 1–2 (14 patients, 35%) or step 3–4 (26 patients, 65%); 12 patients (30%) had at least one steroid course in the year before enrollment. They exhibited normal psychological adjustment but elevated levels of general (U = 179,500; *p* < 0.001) and state anxiety (U = 170,000; *p* < 0.001) compared to healthy peers. Additionally, the asthmatic children’s mothers displayed higher levels of separation anxiety compared to the fathers (t = −2.865, *p* = 0.006). Increased separation anxiety in the mothers corresponded to a history of at least one exacerbation in the previous year. **Conclusions**: The asthmatic children exhibited normal psychological adjustment with higher general and state anxiety. Also, their mothers showed greater levels of separation anxiety compared to their fathers. Lastly, higher mothers’ separation anxiety corresponded to a history of at least one exacerbation in the previous year. The influence of the parents’ psychological well-being on the children’s asthma control was previously unexplored.

## 1. Introduction

Asthma is a chronic inflammatory disease characterized by bronchial hyperreactivity, mucus overproduction, and airway remodeling. According to the Global Initiative for Asthma (GINA) [1], asthma control is considered the goal of asthma treatment mainly because well-treated patients are expected to have minimal chronic symptoms and exacerbations, with no emergency visits and no limitation of daily activities. On the contrary, poorly controlled asthma generates a high socio-economic burden defined in terms of costs to the patient and society. Many factors are involved in unsatisfactory asthma control, including lack of adherence to therapy [2] and emotional disorders [3]. The literature suggests a greater prevalence of emotional and behavioral issues in children with asthma compared to healthy children [4,5,6,7], including elevated rates of internalizing disorders, particularly anxiety [3,8] and separation anxiety [9]. Recent studies have linked children’s anxiety and depression to frequent asthma exacerbations and poorer asthma control [10]. Additionally, caregivers of asthmatic children are more likely to experience anxiety and depressive disorders than caregivers of healthy children [11], and these emotional issues among caregivers may contribute to suboptimal asthma control in children [12,13]. Caregivers are the primary system for children’s development and adaptation, and, especially in the context of a chronic illness, parents are essential for helping children learn how to manage symptoms and consequences and cope with their emotions. Parents’ well-being is crucial in this process; they must delicately balance between meeting the needs of their child, addressing the concerns of other family members, and fulfilling work commitments; they may struggle to accept the child’s diagnosis and worry about their future well-being [11,14,15]. Despite the commonly held view that asthma is best understood within a family-contextual approach, research has predominantly focused on the adaptation of parents and children separately. Recently, there is a growing shift toward a more integrative perspective, which involves investigating the bidirectional influences between children and their parents [16]. Thus, recent literature has focused on parents and the influence of their psychological well-being on children’s asthma, yielding promising results that suggest that emotional disorders exert important effects on asthmatic children [3]. Nonetheless, only a few studies have separately evaluated the emotional burden of the fathers and mothers of asthmatic children, and no studies assessed the impact of parents’ separation anxiety on asthma-related outcomes.

The current study aims to explore the baseline psychological well-being of asthmatic children compared to healthy controls and to explore psychological functioning in the mothers and fathers of asthmatic children, focusing on the influence of mothers’ and fathers’ anxiety and separation anxiety on children’s asthma.

## 2. Materials and Methods

### 2.1. Study Subjects

In this cross-sectional study, we enrolled children with non-severe asthma who attended the outpatient clinic of the Unit of Pediatric Allergy and Respiratory Medicine between 1 September 2022 and 31 August 2023. Asthma diagnosis was confirmed according to the 2023 GINA guidelines [1]. Exclusion criteria were a history of chronic diseases other than asthma (including psychiatric disorders), severe asthma, and insufficient proficiency in the Italian language.

A group of healthy children and their parents was also recruited using snowball sampling. For them, we scheduled appointments for in-person questionnaire administration. Exclusion criteria were a personal history of chronic diseases (including asthma and psychiatric disorders) and insufficient proficiency in the Italian language.

All parents provided written informed consent to the use of clinical data for research purposes. The study was approved by the Ethics Committee of Padova General Hospital (CESC 5278/AO/22) in accordance with the Declaration of Helsinki. No reward was offered for enrollment. Collected data were anonymized and recorded in a database.

### 2.2. Study Design and Methods

At the evaluation, asthmatic children underwent a full anamnestic investigation, including asthma control assessment according to GINA 2023 [1], so that asthma was classified as controlled (GINAscore = 0), partially controlled (GINAscore = 1–2) or uncontrolled (GINAscore = 3–4), depending on the presence of daytime symptoms, night awakening, need for relievers, and limitation to physical activity. In addition, the Asthma Control Test (childhood-ACT or ACT) [17] was administered, evaluating activity limitation, shortness of breath, night-time symptoms, use of rescue limitation, and patient overall rating of asthma control over the previous four weeks. Higher ACT scores indicate better asthma control. Furthermore, we investigated the occurrence of asthma exacerbations (AEs) during the previous year, defined as a worsening in asthma symptoms requiring a course of at least 3 days of oral steroids. The antiasthma therapy taken was classified according to the treatment steps reported in GINA 2023 [1].

All asthmatic children enrolled underwent spirometry and skin prick tests for the most common aeroallergens.

The psychological assessment of children and parents was based on standardized questionnaires. In addition, parents were asked about their age, education level, and family and work situations. The survey’s completion took approximately 30 min. The questionnaires were administered by PhD candidate and psychologist Silvia Spaggiari, who is a co-author of this paper, after receiving appropriate training. The compilation took place in a silent and separate room in the hospital in order to not interfere with the medical procedures. All questionnaires were administered to all recruited children and parents, each completing their version under the supervision of the researcher. Parents were asked not to interfere with their child’s or the other parent’s completion. The researcher provided assistance in case of difficulties in understanding.

The standardized questionnaires administered were the following:

(1) The Strengths and Difficulties Questionnaire (SDQ) [18] is a behavioral screening questionnaire used to assess the psychological adjustment of children and adolescents. It consists of 25 items, rated on a 3-point Likert scale from 0 “not true” to 2 “certainly true”, which investigate various areas of children’s psychological and behavioral functioning (e.g., “I have many concerns”). The questionnaire encompasses five scales: Emotional Symptoms, Conduct Problems, Hyperactivity–Inattention, Peer Problems, and Prosocial Behavior. The Total Difficulties Score (sdq_tds) is obtained by summing the scores of the first four scales, with scores of 20 and above indicating the clinical range. Higher scores indicate more problems in the subscale domain, except for “Prosocial Behavior”, where higher scores correspond to a greater presence of such conduct. The questionnaire has been validated in Italian and showed good psychometric properties. Cronbach’s α values was α(TDS) = 0.80.
(2) The State-Trait Anxiety Inventory for Children (STAI-C) [19] is a specific tool designed to assess anxiety symptoms in childhood. It comprises two scales: state anxiety, which assesses current anxiety symptoms (e.g., “I feel calm”), and trait anxiety, which evaluates the tendency to react anxiously to a perceived threat (e.g., “I am worried about making mistakes”). The items explore various dimensions of anxiety, including feelings of nervousness, worry, and tension in both the current moment and as a general disposition. Children are required to rate 40 items, with 20 items for each scale, using a 3-point Likert scale (for the state scale, e.g., from “not calm” to “very calm”; for the trait scale from “almost never” to “always”). Higher scores correspond to a higher presence of state or trait anxiety. The questionnaire has been validated in Italian and shows good psychometric properties. Cronbach’s alphas were α(S) = 0.95 for the State scale α(T) = 0.86 for the Trait scale.
(3) The General Health Questionnaire-12 (GHQ-12) [20] is a self-report screening instrument consisting of 12 items designed to assess short-term psychological well-being (anxious and depressive symptoms) experienced in the past 2 weeks. The items explore various aspects of psychological functioning, including feelings of strain, loss of confidence, and difficulties in concentration (e.g., “feeling able to concentrate on what you were doing”). Each item is rated on a 4-point Likert scale, from 0 “more than usual” to 3 “much less than usual”. Distinct cut-off scores categorize responses into three ranges: scores 0 to 14 indicate normal global functioning, scores 15 to 19 suggest psychological distress, and scores 19 to 36 indicate significant distress. The questionnaire has been validated in Italian and demonstrated good psychometric properties. Cronbach’s alpha was 0.80 for the mothers and 0.80 for the fathers.
(4) The State-Trait Anxiety Inventory-Y (STAI-Y) [21] is a self-report tool designed to assess anxiety, specifically state and trait anxiety. As for the children’s version, it comprises 40 items, 20 for each scale (e.g., “I feel calm”; “I am pleased with myself”). Respondents rate each item on a 4-point Likert scale: for the state scale from 1 “not at all” to 4 “very much” and for the trait scale from 1 “almost never” to 4 “nearly always”. Higher scores correspond to a higher presence of state or trait anxiety. The questionnaire has been validated in Italian and showed good psychometric properties. Cronbach’s alphas were α(S) = 0.93 and α(T) = 0.89, respectively, for the State and Trait scales for the mothers, and α(S) = 0.93 and α(T) = 0.91 for the State and Trait scales for the fathers.
(5) Adult Separation Anxiety–27 (ASA-27) [22] evaluates symptoms of separation anxiety one might have experienced in adulthood after the age of 18. The items assess various aspects of separation anxiety, including fears of abandonment, excessive worry about loved ones, and distress when separated from significant others (e.g., “I worry excessively about the well-being of someone close to me”). Respondents provide ratings on a 4-point Likert scale, from “never” to “always”, for each of the 27 self-report items. Higher total scores indicate a greater presence of adult separation anxiety symptoms. The questionnaire has been validated in Italian and demonstrated good psychometric properties. Cronbach’s alpha was α = 0.88 for the mothers and α = 0.91 for the fathers.

### 2.3. Statistical Analysis

Mann–Whitney U tests were performed to evaluate the differences between clinical and control groups in psychological functioning. To compare mothers and fathers of children with asthma, we employed Linear Mixed Models (LMM), using the lme4 Version 1.1.35.1 [23] and lmerTest package [24] in R. We added fixed effects of “parent” and children’s age. The random effect of “couples” was incorporated to account for potential correlations within couples. Lastly, regarding the clinical sample, we used logistic regression models to assess which variables were significantly related to children’s asthma exacerbations in one year due to the binary nature of the outcome variable (0 for “absence of exacerbations”, 1 for “at least one exacerbation”). Nagelkerke [25] and Cox and Snell’s [26] R-squared were used. In both models, the variance inflation factor (VIF) for each variable was lower than 10. The analysis was performed using the SPSS v22.0 software package (SPSS Inc., Chicago, IL, USA) and R software (version 2023.12) [27]. A *p*-value < 0.05 was considered statistically significant.

The power analysis revealed that a sample size of 36 subjects per group, with a significance level of 0.05 and power of 0.8, corresponded to medium effect sizes (0.6).

## 3. Results

A total of 40 asthmatic children (29 males; mean age 12.25 years ± 2.56; age range: 8–18 years old), with both mothers and fathers, were enrolled. Moreover, a control sample of 40 children (29 males; mean age 11.83 years ± 2.29; age range: 8–18 years old), matched by age and gender to the clinical sample, and their respective parents, were recruited (see Table 1 for the parents’ characteristics).

### 3.1. Characteristics of Asthmatic Patients

Forty asthmatic children (M = 12.25 years; SD = 2.56; 29 males) were enrolled, whose characteristics are listed in Table 2. The majority of the children (90% with GINA score and 77.5% with ACT score) had well-controlled or partially controlled asthma (see Table 2). Twelve patients (30%) had at least one steroid course in the last year before enrollment (see Table 2). Fourteen patients (35%) were treated according to GINA step 1–2, while 26 patients (65%) according to GINA step 3–4.

### 3.2. Psychological Functioning

The majority of the children in the clinical group (94.6%) scored in the normal range regarding psychological adjustment (SDQ total score). Nonetheless, 86.8% of them reported clinical levels in state anxiety and 21.1% in trait anxiety.

Among the mothers of the asthmatic children, 22.5% showed significant psychological distress in terms of their general well-being. Additionally, 30.8% reported clinical levels of separation anxiety, and 28.2% and 18.4% scored in the clinical range for state and trait anxiety, respectively.

Regarding the fathers of the asthmatic children, 13.2% reported significant psychological distress. Furthermore, 10.3%, 21.2%, and 5.1% of them scored in the clinical range for separation anxiety, state anxiety, and trait anxiety, respectively.

### 3.3. Comparisons between Clinical and Control Groups

The children in the clinical and control groups did not differ in terms of their psychological adjustment (*p* > 0.05). However, the children with asthma demonstrated higher levels of general and state anxiety compared to their healthy peers.

No significant differences (*p* > 0.05) emerged between the mothers of the clinical and control groups as to their psychological well-being, anxiety, and separation anxiety. The same was found with the fathers (see Table 3).

### 3.4. Comparisons between Mothers and Fathers of Children with Asthma in Psychological Well-Being, Anxiety and Separation Anxiety

When it comes to the mothers’ and fathers’ psychological well-being, the LMM did not show any significant main effect of “parent” on GHQ_TOT (beta = 0.3402, t = 0.564, *p* = 0.576). The mothers (M = 16.62; SD = 3.71) and fathers (M = 17.03; SD = 2.82) scored similar in psychological well-being.

The same result emerged regarding anxiety: no significant effect of “parent” on STAI_TOT was detected (beta = −6.8476, t = −1.856, *p* = 0.0713). The mothers (M = 78.65; SD = 17.75) and fathers (M = 71.68; SD = 18.88) showed similar levels of anxiety.

Regarding separation anxiety, we found a significant main effect of the variable “parent” on ASA_TOT (beta = −4.8199, t = −2.865, *p* = 0,006). More specifically, the mothers showed greater levels of separation anxiety (M = 19.74, SD = 9.95) compared to fathers (M = 14.97, SD = 8.14). The R-squared was 0.39: the model effectively captures a substantial portion of the observed variability in separation anxiety responses between the mothers and the fathers.

### 3.5. Logistic Regression Models for Children’s Asthma Exacerbations

The first logistic regression model involving the mothers was statistically significant overall and explained 55% of the variance of the dependent variable (Nagelkerke R^2^). Also, Cox and Snell’s R-squared confirmed a good model fit. The most impactful variables were the children’s age and the mothers’ separation anxiety: lower children’s age and higher mothers’ separation anxiety scores correlated with a history of at least one exacerbation in the previous year (see Table 4).

The second model focused on the fathers. Overall, it was statistically significant and explained the 32% of the variance of the dependent variable (Nagelkerke R^2^). Also, Cox and Snell’s R-squared confirmed a good model fit, even if it was lower when compared to the previous model. In this case, only the children’s age was significantly related to a history of at least one exacerbation in the previous year (see Table 5).

## 4. Discussion

This study analyzed 40 children with non-severe asthma, mainly in good clinical control, and their parents. We mainly focused on psychological functioning and separation anxiety. Psychological functioning encompasses the overall emotional, cognitive, and behavioral health of an individual. The population of children with asthma and their parents are at higher risk of experiencing problems with psychological functioning, which must be taken into account, particularly in a multidisciplinary approach to the disease [4,5,6,7,11]. Separation anxiety, as defined by the DSM-V, is an intense fear of being separated from close attachment figures and a deep concern about their safety and whereabouts [28]. Regarding parents, separation anxiety is a structural variable that develops throughout the caregiver’s life, regardless of the presence of asthma in the child [29]. Separation anxiety not only affects the general psychological well-being of children and their caregivers but may also be a relevant factor in cases of asthma, as symptoms are sudden and frightening and may require parental intervention and presence. Moreover, it is a very new and understudied construct in the field of childhood chronic illnesses.

We demonstrated that the asthmatic children have normal psychological adjustment while showing higher levels of general and state anxiety compared to their healthy peers. We also found greater levels of separation anxiety in the mothers of the children with asthma compared to the fathers. No significant differences in psychological well-being, anxiety, and separation anxiety emerged when comparing either the mothers or the fathers of the clinical and control groups. Lastly, an increase in the mothers’ separation anxiety scores was associated with the children’s history of at least one asthma exacerbation in the previous year.

Among the 40 asthmatic children enrolled (age 8–18 years old), the majority had good clinical control. These children received asthmatic maintenance therapy according to GINA therapy steps 1–2 (14 patients, 35%) or GINA therapy steps 3–4 (26 patients, 65%); most of them had experienced no exacerbation in the year before enrollment. We excluded patients with severe asthma due to specific characteristics of this population [30,31]. The enrolled children have normal spirometric values, as commonly seen in asthmatic children outside of exacerbations.

We enrolled 40 triads of asthmatic children with both mothers (mean age 45.45 y ± 4.06) and fathers (mean age 48.25 y ± 6.13) and 40 triads of healthy children with both mothers (mean age 45.68 y + 5.33) and fathers (mean age 48.83 y ± 5.93). In both the mothers and the fathers of the asthmatic children and controls, more than 90% of the fathers have a full-time job, while only 38–50% of the mothers have full-time jobs, with 7.7–25% of the mothers being housewives/unemployed. Existing literature indicates that the mothers of the asthmatic children are less inclined to work either full-time or part-time, also influenced by factors such as child well-being and the severity of the disease. With the limit of the low sample size in our study, this conclusion is not confirmed. In the asthmatic group, a higher percentage of mothers are employed full-time compared to the control group (48.7% vs. 37.5%), suggesting the influence of other factors in these percentages, such as sociocultural ones [32].

Analyzing psychological well-being, we find that the asthmatic children showed normal psychological adjustment (94.6% have normal SDQ total score), while they had higher levels of general anxiety, as well as state anxiety (86.8%), compared to their healthy peers. Children with mild or moderate asthma tend not to experience significant psychological difficulties compared to their healthy peers [7]. However, anxiety is often associated with asthma due to frightening and unpredictable symptoms [3,8]. Additionally, higher levels of state anxiety observed in the asthmatic children may be attributed to data collection timing, which occurred after medical visits and in hospital settings.

The parents of the asthmatic children appear to mostly have non-pathological psychological functioning, with 77.5% of the mothers and 86.6% of the fathers reporting no psychological distress in terms of their general well-being. Additionally, we described greater levels of separation anxiety in the mothers of the asthmatic children (30.8%) compared to the fathers (10.8%), without any other significant differences in the score of state and trait anxiety. The limited literature indicates that mothers, as primary caregivers, typically experience lower psychological well-being [33,34]. Notably, this study reveals a higher incidence of separation anxiety symptoms in mothers, likely triggered by concerns about complications and the need to manage their child’s respiratory symptoms effectively, especially when not close [35].

In the comparison between the parents of the asthmatic children and the ones of healthy control, we showed similar scores in overall psychological well-being, suggesting that our sample is not psychopathological.

Through logistic regression analysis, we observed that an increase in the mothers’ separation anxiety scores was associated with the history of at least one exacerbation in the previous year, a trend not observed in the fathers. For speculative purposes, we could hypothesize that the mothers with higher separation anxiety scores may exhibit heightened concern regarding their children’s asthma symptoms, which may potentially result in an overemphasis on their children’s symptoms, thus prompting more frequent medical attention, which could contribute to more exacerbations. Additionally, the mothers in our clinical sample were less likely to have full-time jobs compared to the fathers, indicating a potentially greater involvement in managing their children’s illnesses. Furthermore, heightened separation anxiety in the mothers may trigger the fear that their children could experience a worsening of symptoms when away from them. Similar results were found when considering parents’ general anxiety and depression, which may impact asthma severity and control [3]. However, regarding specifically parents’ separation anxiety symptoms and asthma-related outcomes, no studies have been published.

Considering the results of our study, it is expected that a future deeper investigation into the parents’ separation anxiety will hopefully focuses on its association with asthma control. Moreover, given the gap in the literature, we hope that future research could focus on the role of the fathers’ emotional problems on the asthmatic children.

Looking at the limits of our study, first, we intentionally excluded the children with severe asthma, mainly because of the specific characteristics of this condition, the frequent hospitalizations, and invasive procedures. Moreover, the children were enrolled during outpatient control assessment, thus not in a home environment, which could be more familiar and quieter. Then, we only enrolled members of the Caucasian population, as the language barrier prevented us from enrolling the children and parents who were not proficient in the Italian language. Furthermore, it has to be considered that the variable measuring anxiety in the regression model includes a situational component related to the specific moment of the assessment. Lastly, we used self-report questionnaires, which might not accurately reflect participants’ thoughts due to influences of social desirability or misinterpretations of questions.

Our study may expand the analysis regarding our population by not only measuring the situational component of anxiety during the assessment but also analyzing the overall anxiety traits of the asthmatic children and their parents to better understand its impact.

Furthermore, employing alternative methods to self-report questionnaires, such as observational or interview-based techniques, could mitigate the biases introduced by misinterpretations of questions, leading to more accurate analysis.

Lastly, future studies regarding the psychological well-being of children with asthma could be focused on children with severe asthma in order to explore the psychological functioning of this population, probably influenced by frequent hospitalizations and invasive procedures.

## 5. Conclusions

In conclusion, the asthmatic children enrolled in our study had a normal psychological adjustment with higher levels of general and state anxiety. Also, greater levels of separation anxiety were described in the mothers of the asthmatic children compared to the fathers, while no differences in psychological well-being emerged between the mothers and the fathers of the clinical and control groups.

We provided evidence of an increase in the mothers’ separation anxiety scores, which corresponded to a history of at least one exacerbation in the previous year. Indeed, this approach to how parents’ psychological well-being can influence children’s asthma control was previously unexplored.

## Figures and Tables

**Table 1:** Sociodemographic characteristics of the parents’ samples (40 triads).

	CLINICAL GROUP				CONTROL GROUP			
CAREGIVERS	MOTHERS (*n* = 40)		FATHERS (*n* = 40)		MOTHERS (*n* = 40)		FATHERS (*n* = 40)	
	Mean	SD	Mean	SD	Mean	SD	Mean	SD
Age	45.45	4.06	48.25	6.13	45.68	5.33	48.83	5.92
Working situation	N (%)		N (%)		N (%)		N (%)	
Full-time job off-site	19 (48.7%)		38 (97.4%)		15 (37.5%)		27 (90%)	
Part-time job off-site	16 (41.0%)		0 (0%)		12 (30%)		0 (0%)	
Part-time remote work from home	0 (0%)		0 (0%)		1 (2.5%)		0 (0%)	
Full-time remote work from home	1 (2.6%)		1 (2.6%)		2 (5%)		3 (10%)	
Housewife/	3 (7.7%)		0 (0%)		10 (25%)		0 (0%)	
unemployed								
Education level								
Middle school Diploma	7 (17.5%)		10 (25%)		-		-	
High school Diploma	21 (52.5%)		18 (45%)		-		-	
Bachelor’s degree	12 (30.0%)		12 (30%)		-		-	
Family situation								
Married	36 (90%)		36 (90%)		-		-	
Cohabiting	2 (5%)		1 (2.5%)		-		-	
Unofficially separated	1 (2.5%)		2 (5.0%)		-		-	
Divorced	1 (2.5%)		1 (2.5%)		-		-	

**Table 2:** Characteristics of asthmatic children. GINA = Global Initiative for Asthma; ACT = Asthma Control Test; FEV1 = Forced Expiratory Volume in the first second; FVC = Forced Vital Capacity.

Characteristics	Asthmatic Patients
Disease duration (years), mean (SD)	7 (±3.1)
Disease duration (years), range	1–13
Allergic to inhalants, n (%)	37 (92.5%)
Comorbidities	
Allergic rhinitis	27 (67.5%)
Food allergy	14 (35%)
Atopic dermatitis	5 (12.5%)
Maintenance Therapy according to GINA steps	
GINA step 1	11 (27.5%)
GINA step 2	3 (7.5%)
GINA step 3	14 (35%)
GINA step 4	12 (30%)
Asthma control according to GINA score	
GINA score 0—well-controlled	24 (60%)
GINA score 1–2—partially controlled	12 (30%)
GINA score 3–4—uncontrolled	4 (10%)
Asthma control according to ACT score	
ACT score 25 or more—well-controlled	6 (15%)
ACT score 20–24—partially controlled	25 (62.5%)
ACT score less or equal to 19—uncontrolled	9 (22.5%)
Patients with at least 1 steroid course in the last year, n (%)	12 (30%)
Spirometric values	
FEV1 (% pred), mean (SD)	95 (14.9)
FEV1 (z-score), mean (SD)	−0.451 (1.273)
FEV/FVC (% pred), mean (SD)	100 (7.9)
FEV1/FVC (z-score), mean (SD)	0.083 (1.082)
Number of asthma exacerbations in the last year	
None	28 (70%)
At least one	12 (30%)

**Table 3:** Mann–Whitney U Tests to assess differences between clinical and control groups in psychological functioning. Bold for *p* < 0.005. SDQ_TDS = Strengths and Difficulties Questionnaire_Total Difficulties Score; STAI_STATE = State-Trait Anxiety Inventory_state scale; STAI_TRAIT = State-Trait Anxiety Inventory_trait scale; STAI_TOT = State-Trait Anxiety Inventory_total; GHQ_TOT = General Health Questionnaire_total; ASA_TOT = Adult Separation Anxiety_total.

CHILDREN				
Variables	Clinical sample	Control sample	U	*p*
	Average rank	Average rank		
SDQ_TDS	37.43	38.55	682.000	0.824
STAI_STATE	53.03	23.97	170.000	<0.001
STAI_TRAIT	38.75	38.25	712.500	0.921
STAI_TOT	51.51	24.22	179.500	<0.001
MOTHERS				
Variables	Clinical sample	Control sample	U	*p*
	Average rank	Average rank		
GHQ_TOT	42.71	38.29	711.500	0.392
STAI_STATE	42.03	38.03	701.000	0.438
STAI_TRAIT	41.84	37.28	671.000	0.373
STAI_TOT	41.05	37.10	664.000	0.438
ASA_TOT	37.27	32.05	749.500	0.765
FATHERS				
Variables	Clinical sample	Control sample	U	*p*
	Average rank	Average rank		
GHQ_TOT	35.05	33.80	549.000	0.791
STAI_STATE	33.22	36.12	521.500	0.548
STAI_TRAIT	35.28	34.63	574.000	0.894
STAI_TOT	33.70	35.52	539.500	0.706
ASA_TOT	37.27	32.05	496.500	0.283

**Table 4:** Logistic regression model of children’s asthma exacerbations in one year. Covariates considered: children’s age, mothers’ separation anxiety (ASA_TOT), and anxiety (STAI_TOT). (B, unstandardized beta; OR, odds ratio; CI, confidence intervals; std. β, standardized beta; Adj. R2, adjusted R2. Bold for *p* < 0.005). ASA_TOT = Adult Separation Anxiety_total; STAI_TOT = State-Trait Anxiety Inventory_total.

	Asthma Exacerbations in One Year				
	B	OR (95% CI)	Std. β	*t*	*p*
Intercept	10.454	34,692.053 (0.432; 20.476)	−1.659	2.044	0.041
Children’s age (years)	−0.814	0.443 (−1.421; −0.207)	−2.142	6.902	0.009
Mothers’ separation anxiety (ASA_TOT)	0.156	1.169 (0.001; 0.311)	1.450	1.968	0.049
Mothers’ anxiety (STAI_TOT)	−0.064	0.938 (−0.153; 0.025)	−1.142	−1.418	0.156
Model fit		χ^2^ = 18.217			
		*p* < 0.001			
Nagelkerke R^2^		0.552			
Cox and Snell R^2^		0.389			

**Table 5:** Logistic regression model of children’s asthma exacerbations in one year. Covariates considered: children’s age, fathers’ separation anxiety (ASA_TOT), and anxiety (STAI_TOT). (B, unstandardized beta; OR, odds ratio; CI, confidence intervals; std. β, standardized beta; Adj. R2, adjusted R2. Bold for *p* < 0.005). ASA_TOT = Adult Separation Anxiety_total; STAI_TOT = State-Trait Anxiety Inventory_total.

	Asthma Exacerbations in One Year				
	B	OR (95% CI)	Std. β	*t*	*p*
Intercept	7.550	1901.340 (0.200; 14.901)	−1.165	2.013	0.044
Children’s age (years)	−0.559	0.572 (−1.008; −0.110)	−1.428	−2.439	0.015
Fathers’ separation anxiety (ASA_TOT)	0.009	1.009 (−0.122; 0.140)	0.073	0.132	0.895
Fathers’ anxiety (STAI_TOT)	−0.027	0.974 (−0.086; 0.033)	−0.503	−0.871	0.384
Model fit		χ^2^ = 9.676			
		*p* = 0.022			
Nagelkerke R^2^		0.327			
Cox and Snell R^2^		0.230			

## Data Availability

Data are available on request due to ethical reasons.
